# Host DNA Demethylation Induced by DNMT1 Inhibition Up-Regulates Antiviral OASL Protein during Influenza a Virus Infection

**DOI:** 10.3390/v15081646

**Published:** 2023-07-28

**Authors:** Zhiyan Zhao, Jing Li, Ye Feng, Xiaoping Kang, Yuchang Li, Yuehong Chen, Wei Li, Wenguang Yang, Lu Zhao, Shenghai Huang, Sen Zhang, Tao Jiang

**Affiliations:** 1 School of Basic Medical Sciences, Anhui Medical University, Hefei 230032, China; zzhiyan812@163.com (Z.Z.); shhuang@ahmu.edu.cn (S.H.); 2 State Key Laboratory of Pathogen and Biosecurity, Beijing Institute of Microbiology and Epidemiology, Academy of Military Medical Sciences, Beijing 100071, China; lj-pbs@163.com (J.L.); fengye621@126.com (Y.F.); kangxiaoping@163.com (X.K.); liyuchang66@163.com (Y.L.); chenyuehong.happy@163.com (Y.C.); 18567625401@163.com (W.L.); ywg2106@163.com (W.Y.); zhaolu94264@163.com (L.Z.)

**Keywords:** influenza A virus, DNA methyltransferase 1, miR-142-5p, PI3K/AKT, 2′-5′-oligoadenylate synthetase-like protein

## Abstract

Influenza A virus (IAV) is a leading cause of human respiratory infections and poses a major public health concern. IAV replication can affect the expression of DNA methyltransferases (DNMTs), and the subsequent changes in DNA methylation regulate gene expression and may lead to abnormal gene transcription and translation, yet the underlying mechanisms of virus-induced epigenetic changes from DNA methylation and its role in virus–host interactions remain elusive. Here in this paper, we showed that DNMT1 expression could be suppressed following the inhibition of miR-142-5p or the PI3K/AKT signaling pathway during IAV infection, resulting in demethylation of the promotor region of the 2′-5′-oligoadenylate synthetase-like (OASL) protein and promotion of its expression in A549 cells. OASL expression enhanced RIG-I-mediated interferon induction and then suppressed replication of IAV. Our study elucidated an innate immunity mechanism by which up-regulation of OASL contributes to host antiviral responses via epigenetic modifications in IAV infection, which could provide important insights into the understanding of viral pathogenesis and host antiviral defense.

## 1. Introduction

Influenza is a globally circulating human respiratory infectious disease with recurrent epidemic and pandemic potential, which has caused at least 500,000 deaths worldwide each year since the Spanish Flu pandemic killed more than 50 million people in 1918 [1]. Influenza A virus (IAV) is one of the main pathogens of influenza, which is a negative sense-segmented RNA virus belonging to the family Orthomyxoviridae [2]. Clinical symptoms of IAV infection, including chills, headache, fever, pneumonia and even death, are complex interactions between virus and host factors. Although the IAV does not integrate into the genome of the infected cell, the virus improves its pathogenicity to host by multitudinous mutations [3,4], or efficiently switches off the expression of host cell genes to reduce the host response to viral infection by a complex interplay [5,6]. IAV infection results in expression changes of hundreds of interferon-stimulated genes (ISGs) with antiviral and immunomodulatory functions, such as the human 2′-5′-oligoadenylate synthetase-like (OASL) protein. OASL is a member of the OAS family of proteins, which has no OAS enzyme activity and is widely regarded as a part of the host defense mechanism against viral infection [7]. Studies have shown that the OASL protein can be induced by type I or type II interferon (IFN), while OASL can also be activated by binding to viral nucleic acid [8,9]. During RNA virus infection, OASL functions as an anti-viral protein through enhancing the RIG-I pathway and stimulating IFN α/β production [10].

Epigenetic modification is inherited and affects gene expression without changing the DNA sequence. It includes DNA methylation, noncoding RNAs and histone modifications, among which DNA methylation is the most commonly studied epigenetic modification in humans [11]. DNA methylation primarily functions in transcriptional repression and gene activation and is a biochemical reaction that occurs mainly in cytosine–phosphate–guanine (CpG) dinucleotide islands [12]. DNA methylation is mediated by several different methyltransferases. Mammalian DNA cytosine methyltransferase 1 (DNMT1) can maintain methylation patterns during DNA replication. Once DNMT1 recognizes a hemimethylated CpG dinucleotide in the substrate DNA, it will methylate the CpG site [13]. DNMT3a and DNMT3b generally perform de novo methylation of either unmethylated or hemimethylated DNA. Ten-eleven translocation (TET) proteins counter the activity of DNMTs to cause demethylation [14]. Moreover, studies indicate that activation-induced cytidine deaminase (AICDA) and thymine DNA glycosylase (TDG) are also involved in the demethylation process [15].

It has been shown that viral infection can also cause DNA methylation changes in the genome of viruses or host cells through a variety of direct or indirect pathways to regulate DNMT expression, such as in hepatitis B/C virus, Epstein–Barr virus and IAV infections [16,17,18,19]. The DNA methylation changes will result in abnormal expression of downstream cytokines and promotion of viral infection or host antiviral response during the occurrence of diseases [20]. These outcomes suggest that changes in the DNA methylation status of specific genes during viral infection may have a profound effect on the onset and progression of disease.

In this paper, we aimed to elucidate how IAV infection could regulate the expression of host genes by changing the host DNA methylation, thus further affecting the replication of virus. We provided cellular evidence of close correlations among IAV infection, DNMT1 expression, and *OASL* CpG island demethylation intensity, and further revealed that IAV infection down-regulated DNMT1 transcriptional expression by decreasing p-AKT activity or up-regulating miR-142-5p expression. These outcomes resulted in demethylation of *OASL* CpG islands and ultimately promoted OASL expression, which, in turn, inhibited IAV replication.

## 2. Materials and Methods

### 2.1. Cell Culture and Viruses

Human alveolar epithelial A549 cells (ATCC, CCL-185) and Madin–Darby Canine Kidney (MDCK) cells (ATCC, Manassas, VA, USA, CCL-34) were cultured at 37 °C/5% CO_2_ in Dulbecco’s modified Eagle’s medium (Gibco, Carlsbad, CA, USA) containing 10% fetal bovine serum (Gibco), 1% penicillin-streptomycin and 1% Hepes. H1N1 subtype influenza A virus A/California/07/2009 (CA07), A/PR/8/1934 (PR8) and A/Urumqi/XJ49/2018 (XJ49) were propagated in the allantoic cavity of 10-day-old, specific pathogen-free (SPF) chicken embryos.

### 2.2. Drug Treatment

A549 cells were cultured in 6- and 24-well plates. After the cell density reached about 80–90% confluence, the cell culture medium was replaced with serum-free fresh medium containing different concentrations (0, 2.5, 5, 10 μM) of 5-aza-CdR (Sigma-Aldrich, St. Louis, MO, USA, A3656). The medium was changed every 24 h over a period of 72 h. At the end of treatment, cells were collected for subsequent quantitative real-time PCR (qRT-PCR) and Western blot detection.

A549 cells were plated in 24-well plates. After the cell density reached about 80–90%, the cell culture medium was replaced with fresh medium containing 40 μM LY294002 (MCE, HY-10108) or 30 μM 740 Y-P (MCE, HY-P0175). Subsequent experiments were carried out 48 h later.

### 2.3. Quantitative Real-Time PCR (qRT-PCR)

The PureLink viral RNA/DNA Mini kit (Thermo Fisher Scientific, Waltham, MA, USA) was utilized to extract RNA from cells. QRT-PCR was performed using a One Step TB Green PrimeScript PLUS RT-PCR Kit (TaKaRa, Shiga Prefecture, Kusatsu City, Japan, RR096A) with a Light Cycler 480 II (Roche, Basel, Switzerland). The 2^−ΔΔCT^ method was utilized for the assessment of relative gene expression. Utilized primers are listed in Supplementary Table S1.

For miRNA analysis, total RNA was extracted from cells using a miRcute miRNA Isolation Kit (TIANGEN). RNA was reversely transcribed using a miRcute Plus miRNA First-Strand cDNA Kit (TIANGEN) and qRT-PCR was performed using a miRcute Plus miRNA qPCR Kit and miRNA-specific primers (TIANGEN). The expression of miRNA was normalized by the endogenous control U6 snRNA. Each sample needed three replicates. The 2^−ΔΔCT^ method was used to calculate the relative expression.

### 2.4. Plasmids, siRNAs and Other Reagents

A human OASL natural ORF mammalian expression plasmid (pCMV3-OASL) was purchased from Sino Biological Inc. (HG15697-UT, Shanghai, China). The siRNAs, including si-DNMT1, si-OASL and si-ctrl, were synthesized by Sangon Biotech, Shanghai, China (see Supplementary Table S1 for sequences). Hsa-miR-142-5p mimic (V0325) and miRNA Mimic Negative Control (R0516) were purchased from RIBOBIO.

### 2.5. Western Blot Analysis and Antibodies

RIPA Buffer (Thermo Fisher Scientific) containing 10% PMSF (Biomed, London, UK) was used to extract protein from cells, after which a BCA Kit (Sangon Biotech) was employed to measure protein concentrations. Protein samples were separated by electrophoresis on 4–15% (*w*/*v*) SDS-PAGE gels and electrotransferred to polyvinylidene fluoride (PVDF) membranes, followed by blocking for 2 h with 5% fat-free milk at room temperature under agitation. Then the membranes were first incubated with specific primary antibodies overnight at 4 °C and next with horseradish peroxidase (HRP)-conjugated secondary antibodies. Proteins were detected using SuperSignal West Femto’s highest sensitivity chemiluminescence substrate kit (Thermo Science, New York, NY, USA). Images were obtained using the System GelDoc XR+ Imagela (Bio-Rad, Hercules, CA, USA). The gray values of bands in three independent biological repeated experiments were calculated by ImageJ 1.53k software, with following procedures related to removing the influence of background, setting appropriate measurement parameters, setting the unit, reversing the band color and selecting the band to be measured.

The following antibodies were used: rabbit anti-DNMT1 polyclonal (GTX116011, Gene Tex, Irvine, CA, USA), rabbit anti-OASL polyclonal (ab229136, Abcam, Cambridge, UK), rabbit anti-AKT monoclonal (C67E7, CST), rabbit anti-p-AKT monoclonal (Ser473, CST), rabbit anti IAV H1N1 HA polyclonal (GTX127357, Gene Tex), mouse anti-β-actin monoclonal (ab8226, Abcam) and HRP-conjugated secondary antibodies (ZSJQ-BIO; ZB2305/ZB2301).

### 2.6. DNMT Enzymatic Activity Assay

DNMT enzymatic activity was determined using the EpiQuikTM nuclear Extraction kit I (OP-0002, Epigentek, Suffolk County, NY, USA) to extract nuclear extracts from CA07-treated A549 cells. Protein concentrations of nuclear extracts were determined using the BCA kit (Sangon Biotech). DNMT enzymatic activity was measured using an EpiQuik™ DNA Methyltransferase Activity/Inhibition Assay Kit (P-3001, Epigentek). The absorbance was measured at 450 nm using a microplate reader, and DNMT enzymatic activity was calculated with the following formula: DNMT activity (OD/h/mg) = (sample OD–blank OD) × 1000/[Protein Amount (µg) × incubation time (h)].

### 2.7. DNA Methylation Analysis

The bisulfite conversion and sequencing method was performed to measure methylation of the promoter region within genes before and after the treatment of CA07. Genomic DNA from A549 cells was purified using a PureLink™ Genomic DNA Mini Kit (Thermo Fisher Scientific) according to the manufacturer’s instructions. Genomic DNA was treated with the EZ DNA Methylation-Direct™ Kit (Zymo Research, Irvine, CA, USA) to convert unmethylated cytosines into thymines, while preserving methylated cytosines. After bisulfite treatment, the modified DNA was amplified by polymerase chain reaction (PCR). The sequences of the primers for BSP are listed in Supplementary Table S1, and the PCR products were cloned into pEASY-T5 Zero vector (TRAN). Methylation levels were analyzed by sequencing and the results of all nine fragments were integrated.

### 2.8. Immunofluorescence Staining

A549 cells were treated with 40 μM LY294002 or CA07 (MOI = 5) for 48 h. Cells were fixed with 4% pre-cooled formaldehyde for 15 min after removing the media, then followed by washing with PBS once and permeabilized with 0.1% Triton X-100 for 30 min. Next, the cells were blocked in PBS containing 5% bovine serum albumin (BSA) for 1 h at room temperature. After blocking, cells were incubated with anti-NP rabbit polyclonal (GTX125989, GeneTex) and anti-DNMT1 rabbit polyclonal (GTX116011, GeneTex) at 4 °C overnight. Cells were then washed with PBS and incubated with Alexa Fluoro 488-conjugated anti-rabbit antibody (ZF-0511, ZSJQ-BIO) and CY3 (GB21301, Servicebio) for 1 h at room temperature. Lastly, cells were stained by DAPI staining solution for 5 min. The images were captured using an Olympus Fluorescence IX73 Microscope.

### 2.9. GO Annotation and KEGG Enrichment Analysis

The Kyoto Encyclopedia of Genes and Genomes (KEGG) pathway database and the Gene Ontology (GO) database under biological process (BP), cellular component (CC) and molecular function (MF) categories were used for functional annotation and enrichment analyses. The bubble chart for bioinformatics-related data analysis was plotted by http://www.bioinformatics.com.cn (accessed on 23 July 2022), an online platform for data analysis and visualization [21].

### 2.10. Statistical Analysis

The GraphPad Prism 9.0 software was used to conduct all statistical analysis. Two groups were analyzed using Student’s *t*-test. For three sets of experiments and multiple independent experimental data, one-way ANOVA was used when the data conformed to Gaussian distribution; otherwise, a nonparametric test was used. Significance levels were set as follows: * *p* < 0.05; ** *p* < 0.01; *** *p* < 0.001; **** *p* < 0.0001.

## 3. Results

### 3.1. DNMT1 Expression Was Down-Regulated during IAV Infection

**Figure 1:**
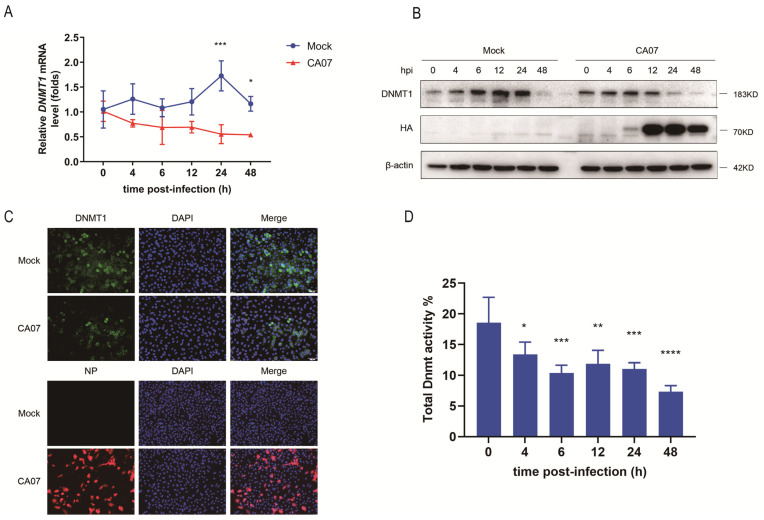
DNMT1 expression was down-regulated during IAV infection. (**A**,**B**) A549 cells were infected with CA07 (MOI = 5). Cells were harvested at the indicated time points (0, 4, 6, 12, 24 and 48 h) and the expression level of DNMT1 was assayed by qRT-PCR (**A**) and Western blot (**B**). The expression level of the influenza A virus HA protein was also detected by Western blot as an infection marker (**B**). (**C**) Immunofluorescence staining showed the localization of DNMT1 in CA07-infected or uninfected cells. A549 cells were mock-infected or infected with CA07 for 48 h. Nuclei were stained with DAPI (blue), DNMT1 was stained green and influenza A virus NP protein was stained red. Images were captured and analyzed by Olympus Fluorescence IX73 Microscope. Representative images are shown. (**D**) A549 cells were infected with CA07 (MOI = 5). Cells were harvested at the indicated time points (0, 4, 6, 12, 24 and 48 h) and the DNMT activity of whole-cell lysates were assayed. Data are presented as the mean ± SD. Significance was calculated using one-way ANOVA with multiple comparison tests. * *p* < 0.05; ** *p* < 0.01; *** *p* < 0.001; **** *p* < 0.0001.

In our previous study, we had found that IAV (H5N1) infection could affect host DNA methylation [22]. To further investigate whether H1N1 infection could also affect DNA methylation in host cells, we first detected expression of *DNMT1*, *DNMT3a*, *DNMT3b* and other related genes (such as the TET protein family and *UHRF1*) in A549 cells infected with three different subtypes of IAV—A/California/07/2009 (H1N1), A/Urumqi/XJ49/2018 (H1N1) and A/PR/8/34 (H1N1)—at different time points (0, 6, 12, 24 and 48 h) by qRT-PCR. The results showed that the expression of *DNMT1* was down-regulated 24 h post-infection for all three IAV infections, and the differences were significant during CA07 (1/3 expression level of the mock group) and PR8 (1/2 expression level of the mock group) infection (Supplementary Figure S1C). At 48 h post-infection, the difference in the CA07 group was still significant (Figure 1A and Supplementary Figure S1C), with the expression level being about 1/2 of the mock group. Further, the expression levels of *DNMT3A* and *DNMT3B* were also decreased compared with the mock group, which was consistent with the results of previous studies [23,24,25]. These results meant that DNMT1 might be another important epigenetic regulatory protein during IAV infection. Meanwhile, we also detected the titers of CA07 in A549 cells at different time points through the expression of *HA* mRNA, and confirmed that CA07 could indeed proliferate effectively in A549 cells, with a final titer of about 10^6^ pfu/mL (Supplementary Figure S1A). In order to better understand the potential mechanisms, we chose CA07 for further analysis, which had a more obvious down-regulation effect on DNMT1. Next, different MOIs (1, 2, 3 and 5) have been used to verify whether the inhibition of DNMT1 was related to the initial infection dose of IAV. It showed that there were no significant differences between the experimental and mock group at MOI = 1 and 2. At MOI = 3, the expression of *DNMT1* tended to decrease, and there was a significant difference 24 h post-infection. However, the expression of *DNMT1* continued to decrease, and the differences were statistically significant at other time points (4 h, 12 h, 24 h and 48 h) at MOI = 5 (Supplementary Figure S1B). Therefore, in order to ensure that all cells could be infected as early as possible, MOI = 5 was selected for further experiments. The Western blot (with HA as an infection marker) and qRT-PCR analyses that followed confirmed that CA07 infection decreased the DNMT1 protein level in A549 cells and that the difference was significant compared with the control group (*p* < 0.05) (Figure 1A,B). However, the expression of DNMT1 also introduced slightly dynamic changes in the mock group, and we speculated that this might be closely related to cell state, such as a decrease in cell viability, apoptosis or autophagy, in the later stages of normal cell culture. Similarly, further immunofluorescence experiments confirmed the down-regulation of DNMT1 in A549 cells after CA07 infection, with NP as an infection marker. The fluorescence intensity of DNMT1 in the CA07-infected group was weaker than in the mock group under microscopic observation, indicating that CA07 infection inhibited DNMT1 expression (Figure 1C). Then, to further study whether IAV infection would also affect the catalytic activity of DNMTs, the total nuclear proteins were purified at different infection times to detect DNMT enzyme activity. We found that total DNMT activity began to decrease 4 h after infection and reached its lowest level at 48 h, which was about half of the initial activity (Figure 1D). Together, these findings showed that CA07 infection down-regulated DNMT1 expression and inhibited DNMT enzyme activity, which may further lead to DNA demethylation in host cells.

### 3.2. IAV Infection Inhibited DNMT1 Expression by Up-Regulating miR-142-5p

**Figure 2:**
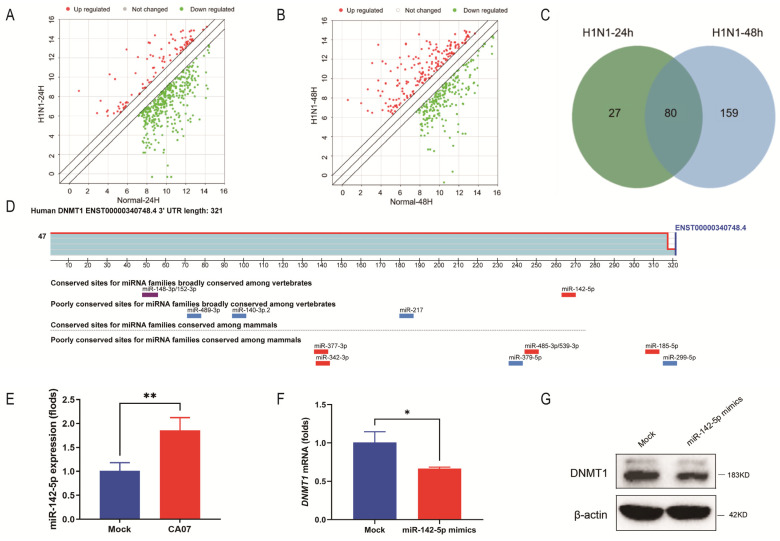
IAV infection inhibited DNMT1 expression by up-regulating miR-142-5p. (**A**,**B**) MiRNA expression after H1N1 infection for 24 h (**A**) or 48 h (**B**) was measured using microarray analysis. Red dots represent up-regulation and green dots represent down-regulation. (**C**) Venn diagram illustrating the number of up-regulated miRNAs and the number of co-up-regulated miRNAs at 24 h and 48 h. (**D**) The binding sites of miR-142-5p to DNMT1 predicted via a bioinformatics website. (**E**) The expression of miR-142-5p after CA07 infection was detected by qRT-PCR. (**F**,**G**) A549 cells were transfected with miR-142-5p mimic or miRNA-NC for 48 h, and the influence of miR-142-5p mimic on the expression of DNMT1 mRNA or protein levels was measured by qRT-PCR (**F**) or Western blot methods (**G**). Data are expressed as the mean + SD of three independent experiments. * *p* < 0.05; ** *p* < 0.01 (Student’s *t*-test).

In our previous study, we performed microarray analysis of miRNAs to investigate the miRNA profile changes during IAV infection [22]. The scatter plots showed the miRNA expression changes after 24 h and 48 h of H1N1 infection, with red representing up-regulation and green representing down-regulation (Figure 2A,B). A Venn diagram (Figure 2C) showed that 80 miRNAs were up-regulated at both 24 h and 48 h post-infection (up-regulated expression change fold ≥ 2). In order to confirm whether there were potential miRNAs that could target *DNMT1* among these 80 candidates, we first used an online tool, TargetScan (https://www.targetscan.org/vert_80/) (accessed on 19 January 2022), to predict whether 12 miRNAs could target *DNMT1* (Figure 2D). Combining the Venn diagram of miRNA profile results and the prediction results showed that miR-142-5p might be a pivotal miRNA in IAV down-regulated DNMT1. In order to further validate this, A549 cells were treated with CA07 for 48 h with MOI = 5, and then the expression of miRNA-142-5p was detected by qRT-PCR. The results showed that the expression of miRNA-142-5p was, significantly, twice as high as that of the mock group after CA07 infection (Figure 2E). To confirm that DNMT1 is a potential target of miR-142-5p, we transfected A549 cells with miRNA-142-5p mimic for 48 h and then measured the abundance of DNMT1. As expected, up-regulation of miRNA-142-5p in A549 cells reduced the DNMT1 mRNA level and protein level to about 60% of the level in the mock group (Figure 2F,G). The data suggested that DNMT1 was the downstream target of miR-142-5p in A549 cells, which could be up-regulated during IAV infection.

### 3.3. IAV Infection Inhibited the PI3K/AKT Signaling Pathway, Resulting in Down-Regulation of DNMT1

**Figure 3:**
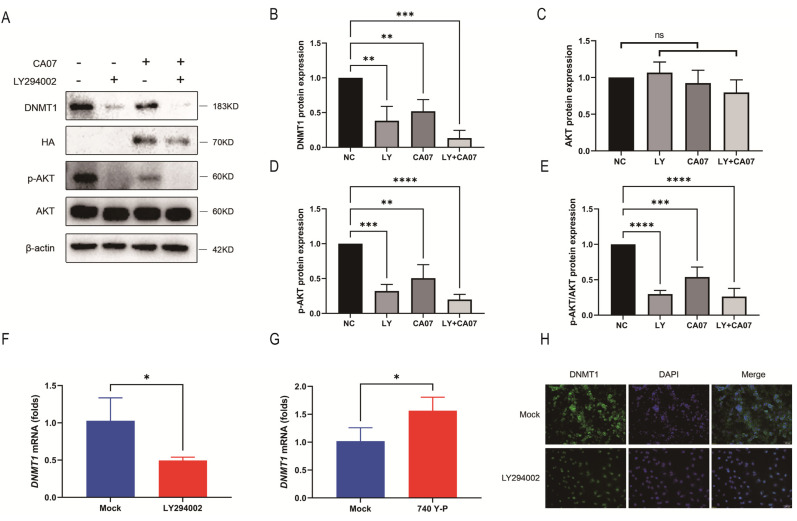
IAV infection inhibited the PI3K/AKT signaling pathway, resulting in down-regulation of DNMT1. (**A**) A549 cells were treated with LY294002 (40 μM) or infected with CA07 (MOI = 5) for 48 h. Cell lysates were subjected to Western blot analysis to measure DNMT1, influenza A virus HA protein, p-AKT and total AKT levels. β-actin was utilized as an internal reference. (**B**–**D**) The gray values of DNMT1 (**B**), AKT (**C**) and p-AKT (**D**) in three independent biological repeated experiments were calculated by ImageJ software. (**E**) The ratio of p-AKT to total AKT was calculated. (**F**,**G**) QRT-PCR detection of *DNMT1* mRNA in A549 cells treated with LY294002 (40 μM) (**F**) or 740 Y-P (30 μM) (**G**). (**H**) A549 cells were treated with DMSO (negative control) or LY294002 (40 μM) for 48 h. DNMT1 expression was visualized by immunofluorescent staining. DNMT1 was stained green; nuclei were stained blue (DAPI). Data are expressed as the mean + SD of three independent experiments. ns, no significance; * *p* < 0.05; ** *p* < 0.01; *** *p* < 0.001; **** *p* < 0.0001 (Student’s *t*-test).

To further verify whether there were other molecular mechanisms for down-regulating DNMT1 in CA07 infections in addition to miRNA-142-5p, we investigated the complex signaling pathways related to IAV infection and epigenetic mechanisms. Studies have shown that the PI3K/AKT signaling pathway may be involved in regulating methylation-related genes, such as DNMT1, DNMT3a or TETs, during differentiation of neural stem cells [26], so we studied the possible role of PI3K/AKT in IAV down-regulation of DNMT1. We first assessed the expression levels of key proteins involved in the PI3K/AKT signaling pathway. The PI3K/AKT signaling pathway could be activated by phosphorylation, also known as p-AKT. The ratio of p-AKT to total AKT is one of the key factors in the activation or inhibition of this signaling pathway. We first detected whether CA07 infection would affect the phosphorylation of AKT. Western blot results revealed that CA07 infection did not change the expression of total AKT (Figure 3A,C), but the amounts of p-AKT were significantly reduced (Figure 3A,D); that is, the proportion of p-AKT in the total AKT was also significantly reduced during CA07 infection (Figure 3E), which meant that the PI3K/AKT signaling pathway was obviously inhibited by IAV infection. Then, A549 cells were treated for 48 h with AKT inhibitor LY294002 and activator 740 Y-P, respectively, and total RNA was purified to analyze the abundance of *DNMT1*. This indicated that the AKT inhibitor could reduce the expression level of DNMT1 to about 50% of the level in the mock group; on the contrary, the activator promoted its expression by 1.5 times (Figure 3F,G). Furthermore, Western blot (Figure 3A,B) and immunofluorescence (Figure 3H) results also indicated a decrease in the DNMT1 expression level (decreased fluorescence) in LY294002-treated A549 cells, indicating the inhibitory effect of LY294002 on the PI3K/AKT signaling pathway could directly lead to a decrease in DNMT1. The inhibitory effect of LY294002 on AKT phosphorylation was similar to that of CA07 infection (Figure 3A,C–E), which further confirmed that CA07 infection inhibited the phosphorylation of AKT. These observations implied that CA07 infection could down-regulate DNMT1 expression by inhibiting the PI3K/AKT signaling pathway independently.

### 3.4. Oxford Nanopore Technologies Sequencing Screened Demethylated Genes

**Figure 4:**
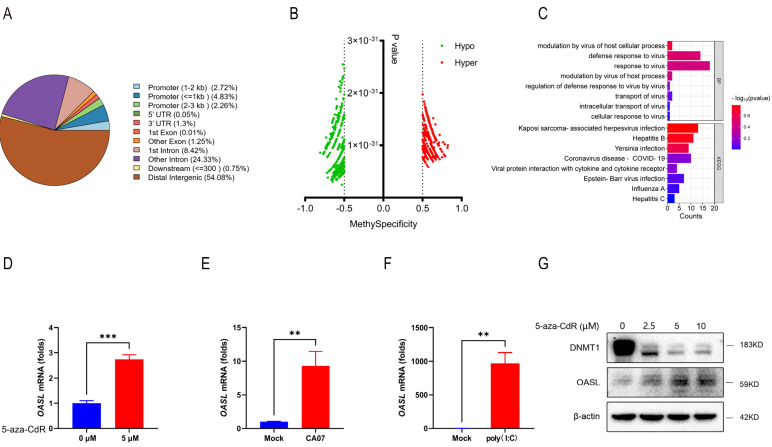
Oxford Nanopore Technologies sequencing screened demethylated genes. (**A**) Classification of differentially methylated genes. (**B**) Scatter plots showing the orientation of the differentially methylated sites. The red plots indicate the hypermethylation genes and the green plots indicate the hypomethylation genes. (**C**) GO and KEGG pathways enrichment analysis of demethylated genes associated with viruses. GO, Gene Ontology; KEGG, Kyoto Encyclopedia of Genes and Genomes. (**D**) A549 cells were treated with 5-aza-CdR at concentrations of 0 and 5 μM for 72 h to detect mRNA expression levels of the screened genes. (**E**) A549 cells were infected with CA07 (MOI = 5) for 48 h to detect mRNA expression levels of the screened genes. (**F**) A549 cells were treated with poly (I:C) for 12 h to detect mRNA expression levels of the screened genes. (**G**) Western blot results of OASL and DNMT1 in A549 cells after treatment with 5-aza-CdR at 0, 2.5, 5 and 10 μM for 72 h. Data are presented as the mean ± SD. Significance was calculated using one-way ANOVA with multiple comparison tests. ** *p* < 0.01; *** *p* < 0.001.

To screen for differentially methylated genes, Oxford Nanopore Technologies (ONT) sequencing was used to detect the whole-genome methylation status of host cells before and after IAV infections. A549 cells were either mock-infected or infected with CA07 for 24 h, and the total genomic DNA were purified for further ONT sequencing. By analyzing the differential methylation sites, we found that the regions with large methylation changes were distal intergenic regions (54.08%), intron regions (32.75%) and promoter regions (9.81%) (Figure 4A). Considering that the promoter regions tended to have a stronger regulatory effect on gene expressions, we further selected the promoter regions for in-depth analysis. Scatter plots showed the orientation of the differentially methylated sites in the promoter regions between mock-infected and CA07-infected A549 cells, with hyper representing hypermethylation and hypo representing hypomethylation (Figure 4B). To screen for demethylated genes associated with IAV infection, we performed functional annotation of these genes. GO analysis of the biological process (BP) showed that there were 23 genes (*POLR3G*, *AICDA*, *TTC4*, *OASL*, *NT5C3A*, *POLR3A*, *SLFN11*, *BNIP3L*, *APOBEC3H*, *ZBP1*, *PLSCR1*, *BECN1*, *IFITM3*, *BNIP3*, *NTRK3*, *CASP8*, *AP2A1*, *SIGLEC1*, *NUP205*, *LGALS8*, *SRC*, *GATA3*, *NPC2*) mainly involved in the interactions between host and IAV infection, including the modulation by virus of the host cellular process, defense response to virus, response to virus, regulation of defense response to virus by virus, transport of virus, intracellular transport of virus and cellular response to virus. The KEGG pathway analysis showed that five genes (*CREBBP*, *HLA-DOB*, *PIK3R2*, *CASP8*, *PRKCB*) were enriched in the signaling pathways of IAV infection (Figure 4C). Subsequently, the expression levels of these selected 27 genes were detected in 5-aza-CdR-treated and CA07-infected A549 cells. 5-aza-CdR is a cytidine analog with a nitrogen atom substituting the carbon in the 5′ position of the heterocyclic ring. It is able to inhibit DNA methylation by reducing the enzymatic activity of DNA methyltransferase via the formation of a stable complex between the enzyme and 5-aza-CdR-substituted DNA. As a consequence, the newly synthesized DNA in exposed cells becomes significantly hypomethylated [27]. The results showed that *ZBP-1*, *OASL*, *HLA-DOB*, *IFITM3* and *NT5C3A* were all increased in different multiples (Figure 4D,E and Supplementary Figure S2A,B). Then we used poly (I:C) as a positive control to screen for any ISG-related genes, and found that *ZBP-1*, *OASL*, *HLA-DOB* and *IFITM3* mRNA expressions were highly correlated with poly (I:C) (Figure 4F and Supplementary Figure S2C). Among them, the expression of *ZBP-1* and *OASL* were significantly up-regulated (Supplementary Figure S2C). ZBP-1 participated in effective host defense pathways that reduce the duration of infection by driving cell death in early infection [28]. OASL is a member of IFN signaling pathways and is often a key antiviral factor induced by IFN. Recent studies have shown that OASL could be a key signaling adapter in the necrotic apoptotic pathway of RIPK3-ZBP-1 after virus invasion and could promote antiviral activity to limit virus replication and transmission [29]. Considering the gene functions, we finally selected *OASL* as the target gene for further exploration in this study. Western blot confirmed that 5-aza-CdR could down-regulate the expression of DNMT1 and up-regulate the expression of OASL (Figure 4G).

### 3.5. The Down-Regulated DNMT1 Expression Caused Demethylation of the OASL Promoter Region during IAV Infection

**Figure 5:**
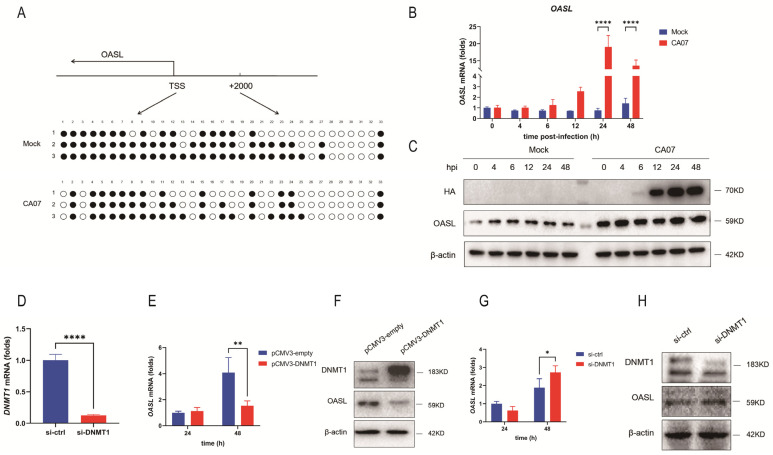
The down-regulated DNMT1 expression caused demethylation of the OASL promoter region during IAV infection. (**A**) Bisulfite sequencing results of the CpG island of the *OASL* promoter region. Black and white circles indicate methylated and unmethylated CpG sites. (**B**,**C**) MRNA (**B**) and protein (**C**) expression levels of OASL in A549 cells infected with CA07, with the influenza A virus HA protein as an infection marker (**C**). (**D**) SiRNA against DNMT1 or si-control were transfected into A549 cells for 48 h and the efficiency of knockdown was measured by qRT-PCR. (**E**,**F**) A549 cells were transfected with the plasmid over-expressing DNMT1 or an empty vector. The expression of OASL was measured by qRT-PCR (**E**) and Western blot (**F**). (**G**,**H**) SiRNA against *DNMT1* was transfected into A549 cells for 48 h and the expressions of OASL were measured by qRT-PCR (**G**) and Western blot (**H**). The empty vector (pCMV3-empty) and si-control served as negative controls. Data are presented as the mean ± SD. Significance was calculated using one-way ANOVA with multiple comparison tests. * *p* < 0.05; ** *p* < 0.01; **** *p* < 0.0001.

To verify the demethylation of the *OASL* promoter region, we performed bisulfite sequencing PCR of CA07 (MOI = 5) virus-infected A549 cells to detect epigenetic changes. Interestingly, infected cells with CA07 virus decreased the percentage of methylated CpGs in the *OASL* promoter region (Figure 5A). Next, we assessed the expression of OASL in IAV-infected A549 cells via qRT-PCR and Western blot. The results showed that IAV infection notably promoted the expression of OASL in A549 cells to nearly 20 times higher than that of the mock group (Figure 5B,C). The results also suggested that IAV infection triggered demethylation of the promoter region, which then caused up-regulation of OASL expression. To obtain more information between the DNA demethylation and OASL up-regulation upon IAV infection, A549 cells were transfected with plasmids expressing DNMT1 (pCMV3-DNMT1) (Figure 5F) or with DNMT1-specific siRNA (si-DNMT1) (Figure 5D,H). At 48 h post-transfection, the qRT-PCR and Western blot analyses showed that expression of exogenous DNMT1 protein reduced expression of OASL to about 40% when compared with cells transfected with empty vectors, whereas si-DNMT1 induced production of OASL to nearly 1.4 times higher than that of the mock group (Figure 5E–H). These observations suggested that OASL was regulated by DNMT1. Taken together, these results indicated that CA07 induced OASL expression by inducing demethylation of the promoter region, which was caused by down-regulation of DNMT1.

### 3.6. OASL Inhibited IAV Replication in A549 Cells

**Figure 6:**
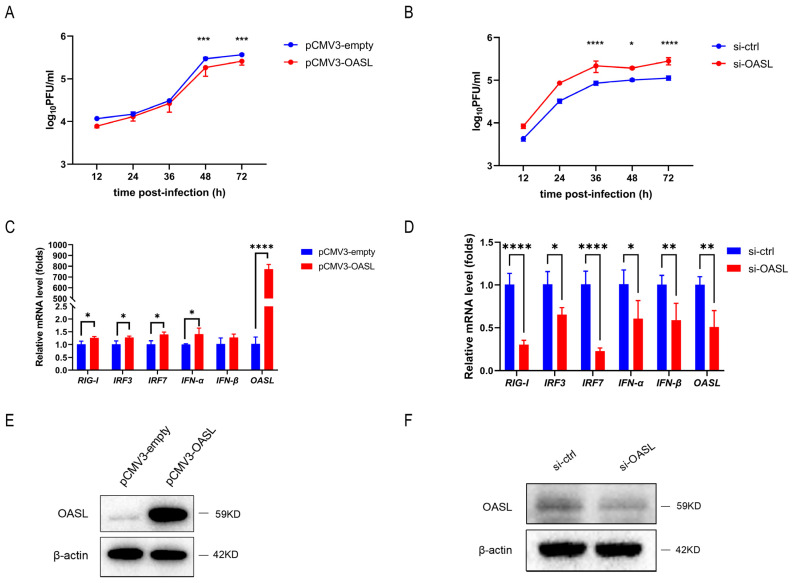
OASL inhibited IAV replication in A549 cells. (**A**,**B**) A549 cells were transfected with plasmids expressing OASL (pCMV3-OASL) (**A**) or si-OASL (**B**), and were subsequently infected with CA07 (MOI = 0.1) at 48 h post-transfection. Virus titer was determined by qRT-PCR and converted to pfu numbers by standard curve. Virus growth curves were examined. (**C**,**D**) A549 cells were transfected with plasmids expressing OASL (pCMV3-OASL) (**C**) or si-OASL (**D**) for 48 h. Total RNA was collected and the mRNA levels of *RIG-I*, *IRF3*, *IRF7*, *IFN-α* and *IFN-β* were analyzed by qRT-PCR. (**E**,**F**) Validation of the transfection efficiency of OASL. The empty vector (pCMV3-empty) and si-control served as negative controls. Data are presented as the mean ± SD. Significance was calculated using one-way ANOVA with multiple comparison tests. * *p* < 0.05; ** *p* < 0.01; *** *p* < 0.001; **** *p* < 0.0001.

To determine whether OASL could affect IAV infection, A549 cells were transfected with plasmids expressing OASL (pCMV3-OASL, Figure 6E) or empty vectors as a control, and were subsequently infected with CA07 (MOI = 0.1) at 48 h post-transfection. Meanwhile, A549 cells were transfected with siRNA-OASL (Figure 6F) or si-ctrl as a control and similarly infected with CA07 viruses. Virus titers were measured at 12, 24, 36, 48 and 72 h post-infection. The results showed that the viruses yielding from cells’ over-expression of OASL were lower than that in the control group (Figure 6A), whereas siRNA-OASL-treated cells produced more IAV particles than wild-type cells (Figure 6B). To further explore the antiviral mechanism of OASL, A549 cells were transfected with pCMV3-OASL or negative control plasmids for 48 h. QRT-PCR data showed that the mRNA levels of *RIG-I*, *IRF3*, *IRF7* and *IFN-α* were increased by 1.2–1.5 times (Figure 6C), which were all important molecules in the host innate immune response. Meanwhile, in the A549 cells, the decreased expression of OASL resulted in the down-regulation of *RIG-I*, *IRF3*, *IRF7* and *IFN-α* in different degrees (Figure 6D). Collectively, these results suggested that OASL could inhibit IAV replication through the RIG-I signaling pathway.

## 4. Discussion

Epigenetic changes induced by IAV infection have been reported in recent years, which could affect the expression of downstream inflammatory factors and immunoreactive cytokines, such as IL-32 [24], cyclooxygenase 2 or IFN-λ1 [23]. Influenza virus infection leads to a decrease in UHRF1-mediated DNA methylation, which increases type I IFN production [30]. In particular, recent studies have also shown that the interaction between IAV nonstructural protein 1 (NS1) and host DNMT3B could cause demethylation of the promoter regions of JAK/STAT signaling pathway inhibitor family members and antagonize the host antiviral immune response [25]. As shown in our previous report, miR-203 was up-regulated by DNA demethylation of the promoter region during H5N1 infection, and inhibited virus replication by targeting the down-regulator of transcription1 (DR1) [22].

Here, we focused mainly on host DNA methylation changes during IAV infection and described a potential mechanism explaining the antiviral activity of OASL via the suppression of DNMT1 by IAV infection. The expression levels of DNA methylation-related genes, *DNMTs*, TET families and some protein cofactors during IAV infection were investigated. The data clearly showed that different H1N1 strains, including PR8/1934, CA07/2009 and XJ49/2019, could all inhibit DNMT1 expression to different degrees, as well as other DNMTs, such as DNMT3A [23] or DNMT3B [24,25], which have been reported. The results showed that IAV infection could not only regulate expression of DNMTs [23], as we have studied, but also affect intracellular transport, post-translational modification [25] or binding ability to the target genes [24] of DNMTs. In our findings, DNMT1 might also be another important epigenetic regulatory protein during IAV infection, in addition to DNMT3A and DNMT3B, which have been explored. Moreover, the data also showed that DNMT1 might be a key enzyme involved in DNMT activity, and its suppression presented an obvious correlation with DNMT activity. Thus, it can be seen that the regulation of IAV infection with respect to DNMTs involves extremely complex interactions, which deserve more in-depth exploration.

MicroRNAs (miRNAs) are 19 to 24 nt non-coding RNAs that regulate gene expression by binding to target messenger RNAs (mRNAs). During viral infections, host miRNAs are involved in various signaling pathways that modulate host–virus interactions [31]. The higher mutation rate of RNA viruses leads to a lower probability of direct binding to miRNAs, so RNA viruses often manipulate the expression levels of specific miRNAs in host cells [32]. The miR-29 family can participate in the regulation of DNA methylation by targeting DNMT3A and DNMT3B in the process of IAV infection [33]. Our data indicated that miR-142-5p expression was up-regulated during IAV infection, which could inhibit DNMT1 expression. In a previous study, miR-142-5p could directly bind to the 3′-UTR region of DNMT1 in breast cancer cells to promote re-expression of the maspin tumor suppressor gene, resulting in inhibition of the migration and proliferation of tumor cells [34]. This was consistent with our conclusion that miR-142-5p targeted DNMT1. In addition, these results meant that miRNAs could play an active role in epigenetic mechanisms, whether in viral infection or tumorigenesis, resulting in adjustable feedback pathways that can fine-tune gene expression. These results are worthy of further attention in future research on epigenetic mechanisms.

There may be other repression mechanisms for the regulation of DNMT1 besides the miRNA pathway. The complex interactions between IAV and the host during infection are often accompanied by changes to signaling pathways, such as the NF-κB/IκB pathway, the MAPK-related signaling pathway, and the PI3K/AKT signaling pathway [35,36]. The PI3K/AKT signaling pathway is involved in the regulation of DNA methylation [37,38]. AKT stabilizes DNMT1 by phosphorylating Ser 143, and dephosphorylation of this site renders DNMT1 susceptible to ubiquitin-mediated proteasomal degradation [39,40]. In our research, we confirmed that activated PI3K/AKT could also directly inhibit DNMT1 mRNA expression at the transcriptional level, which would be an effective supplement to the previous results. The PI3K/AKT signaling pathway has multiple functions in the process of virus infection, including the suppression of apoptosis, synthesis of RNA, alternative splicing, endocytosis and remodeling of actin [41]. Numerous studies have highlighted the different roles of PI3K/AKT signaling in IAV infection. Noriyuki Hirata et al. found that AKT phosphorylation could be induced by PR8 infection in A549 cells (MOI = 0.1) [42]. This was contrary to our results, in which p-AKT could be inhibited by CA07 infection or treatment of inhibitors, such as LY294002. Previous reviews have suggested that PI3K regulation by influenza viruses is a rather complex issue, which varies with different infection times or with different strains (IAV or IBV) [43]. As a result, we speculated that the reasons for the differences might be the different observation periods, the different amounts of viral load or the different strains. In our study, p-AKT was detected 48 h post-infection with MOI = 5 (Figure 3A), while in the research of Noriyuki Hirata et al., the cells were infected at MOI = 0.1 for only 18 h [42]. This meant that the influence on host cellular response may involve dynamic changes. Moreover, PR8 is a relatively old strain, while CA07 was a pandemic strain in 2009, which may cause differences in virulence, pathogenicity or immune response. In addition, the cell culture conditions, such as type of culture medium or the amounts of TPCK-treated trypsin used, might also affect the results. When considering further research, studies exploring these issues would be of great help in further clarifying the regulation of host epigenetics by IAV infection. Other studies have shown that H5N1 IAV infection could induce autophagy by inhibiting the AKT-mTOR pathway, which may provide evidence for our study [44]. The experimental data showed that inhibition of AKT phosphorylation at Ser 473 in A549 cells might be one of the important factors leading to the down-regulation of DNMT1. In conclusion, our study explored the possible roles of viral down-regulation of the PI3K/AKT pathway in the inhibition of DNMT1. In addition, the inhibitory effect of the PI3K/AKT signaling pathway was independent of miR-142-5p, which reflected that IAV infection could regulate DNMT1 simultaneously in different ways through complex interaction networks.

Unlike traditional bisulfite transformation and PCR amplification, nanopore methylation sequencing technology detects DNA modification via the difference in the intensity of current generated by nanopores on unmodified and modified bases with higher resolution [45,46]. The genome-wide methylation status of host cells before and after IAV infection was examined by ONT sequencing, followed by GO analysis, KEGG analysis and experimental verification to screen target genes. By using ONT sequencing, OASL, an innate immune protein, was identified as the target gene. Studies have shown that OASL, IFN-inducing antiviral enzymes, could inhibit the replication of specific RNA viruses, including picornavirus [47], RSV [48], etc. Our study confirmed that OASL could be directly regulated by DNA demethylation, in addition to IFN-inducing, during IAV infection. To determine the effect of up-regulated OASL, CA07 growth curves were constructed in cells transfected with pCMV3-OASL or si-OASL, and the results showed that over-expression of OASL inhibited CA07 replication to a certain extent. OASL acted as an antiviral protein by enhancing the RIG-I signaling pathway in RNA virus infection [8]. The expression levels of *RIG-I*, *IRF3*, *IRF7* and *IFN-α* only slightly increased after over-expression of OASL. We concluded carefully that the up-regulation of OASL could drive the expression of RIG-I, IRF3 and IRF7, which, in turn, promoted the production of IFN-α and exerted its antiviral effect.

## 5. Conclusions

In summary, there is increasing interest in the role of epigenetic modifications in viral infections. We described the complex mechanisms exerted by OASL in its antiviral activity through epigenetic modifications. The modifications were regulated by two independent mechanisms of miRNA-142-5p and the PI3K/AKT signaling pathway. Epigenetic modifications in mammalian cells would affect a variety of biological processes, so we believed that there must be other target genes as well. Moreover, the connections between DNMT1 and virus-induced innate responses must entail a more complex network involving other intermediate molecules. This might be a direction for further research. Our findings provide new insights that improve our understanding of the epigenetic changes in host cells during IAV infections, which confer antiviral signal regulations to combat viral infections.

## Data Availability

Whole-genome methylation sequence data used in this study have been deposited with the China National Microbiology Data Center (NMDC; https://nmdc.cn/en) (accessed on 2 March 2023) under the BioProject accession number: NMDC10018335. MiRNA expression data are available from the GEO database (GSE107186) [22].

## Supplementary Materials

The following supporting information can be downloaded at https://www.mdpi.com/article/10.3390/v15081646/s1: Figure S1, expression of methylation-related genes during IAV infection; Figure S2, experimental verification of screened demethylated genes; Table S1, sequences of primers used in the study.
