# Supplementary material for: Host DNA Demethylation Induced by DNMT1 Inhibition Up-Regulates Antiviral OASL Protein during Influenza a Virus Infection
Source: Viruses. 2023 Jul 28;15(8):1646. doi: 10.3390/v15081646 (PMC10459088; doi:10.3390/v15081646)
Supplement: Supplementary file 1 [file viruses-15-01646-s001.zip › viruses-2514038-supplementary.pdf]

Supplementary Material

1 Supplementary Figures and Table

1.1 Supplementary Figures

Supplementary Figure 1. Expression of methylation-related genes during IAV infection.

(A) A549 cells were infected with A/California/07/2009 (H1N1). Cells were harvested at indicated time points (0, 4, 6, 12, 24 and 48 h) and the expression levels of *HA* mRNA were assayed by qRT-PCR. Then titers of influenza virus were calculated by standard curve. **(B)** A549 cells were infected with A/California/07/2009 at different MOI (1, 2, 3, 5). Cells were harvested at indicated time points (0, 4, 6, 12, 24 and 48 h) and the expression levels of *DNMT*s were assayed by qRT-PCR. **(C-I)** A549 cells were infected with A/California/07/2009 (H1N1), A/Urumqi/XJ49/2018 (H1N1) and A/PR/8/34 (H1N1) (MOI=5), separately. Cells were harvested at indicated time points (0, 6, 12, 24 and 48 h) and the expression levels of *DNMT*s, *TET*s and *UHRF1* were assayed by qRT-PCR. Data are presented as the mean ± SD. Significance was calculated using one-way ANOVA with multiple comparison tests. **p* < 0.05; ***p* < 0.01; ****p* < 0.001; *****p* < 0.0001.

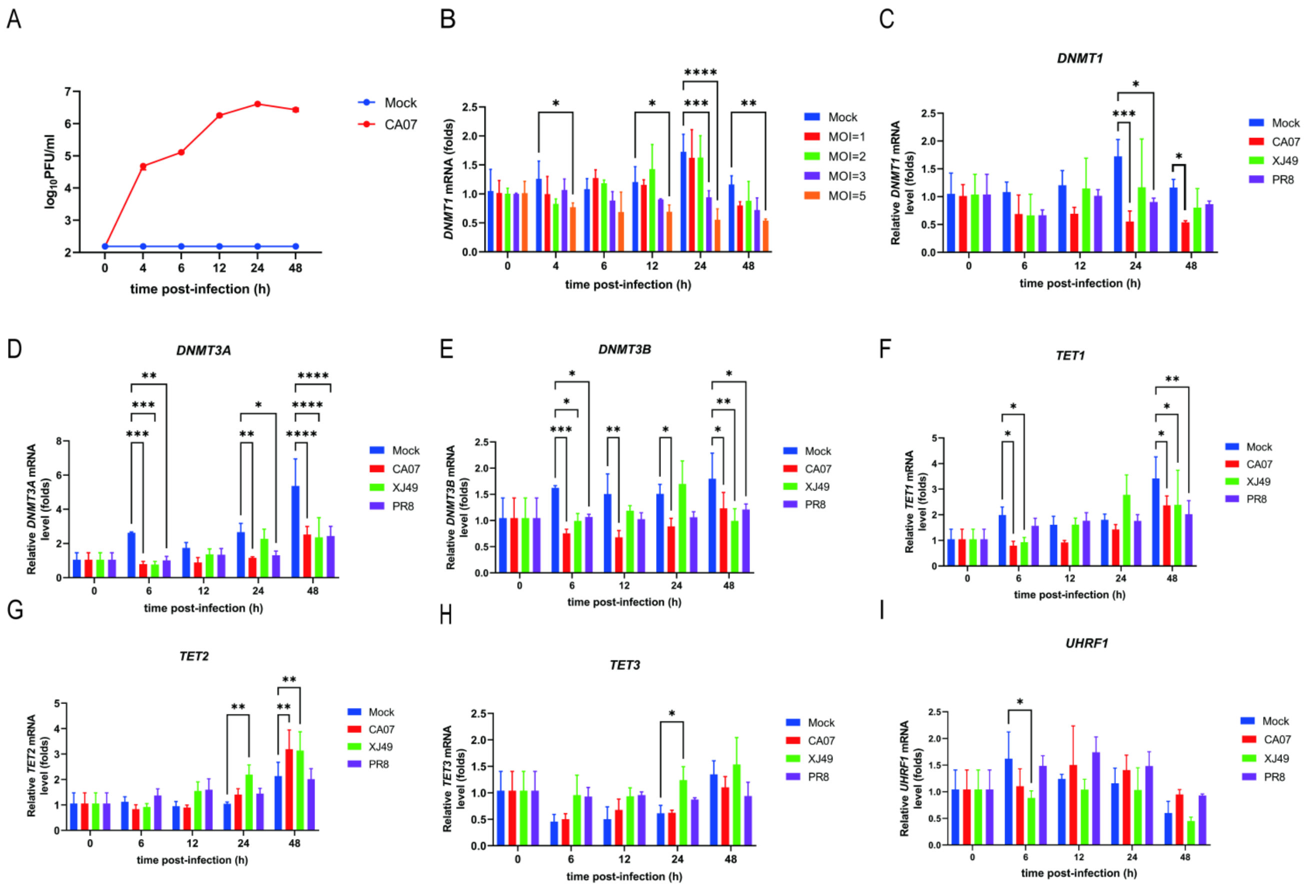

Supplementary Figure 2. Experimental verification of screened demethylated genes.

(A) A549 cells were treated with 5-aza-CdR at concentrations of 0 and 5 μM for 72 h to detect mRNA expression levels of the screened genes. **(B)** A549 cells were infected with CA07 (MOI=5)

for 48 h to detect mRNA expression levels of the screened genes. **(C)** A549 cells were treated with poly (I:C) for 12 h to detect mRNA expression levels of the screened genes. Data are expressed as the mean + SD of three independent experiments. *$p < 0.05$; **$p < 0.01$; ***$p < 0.001$; ****$p < 0.0001$ (Student's *t* test).

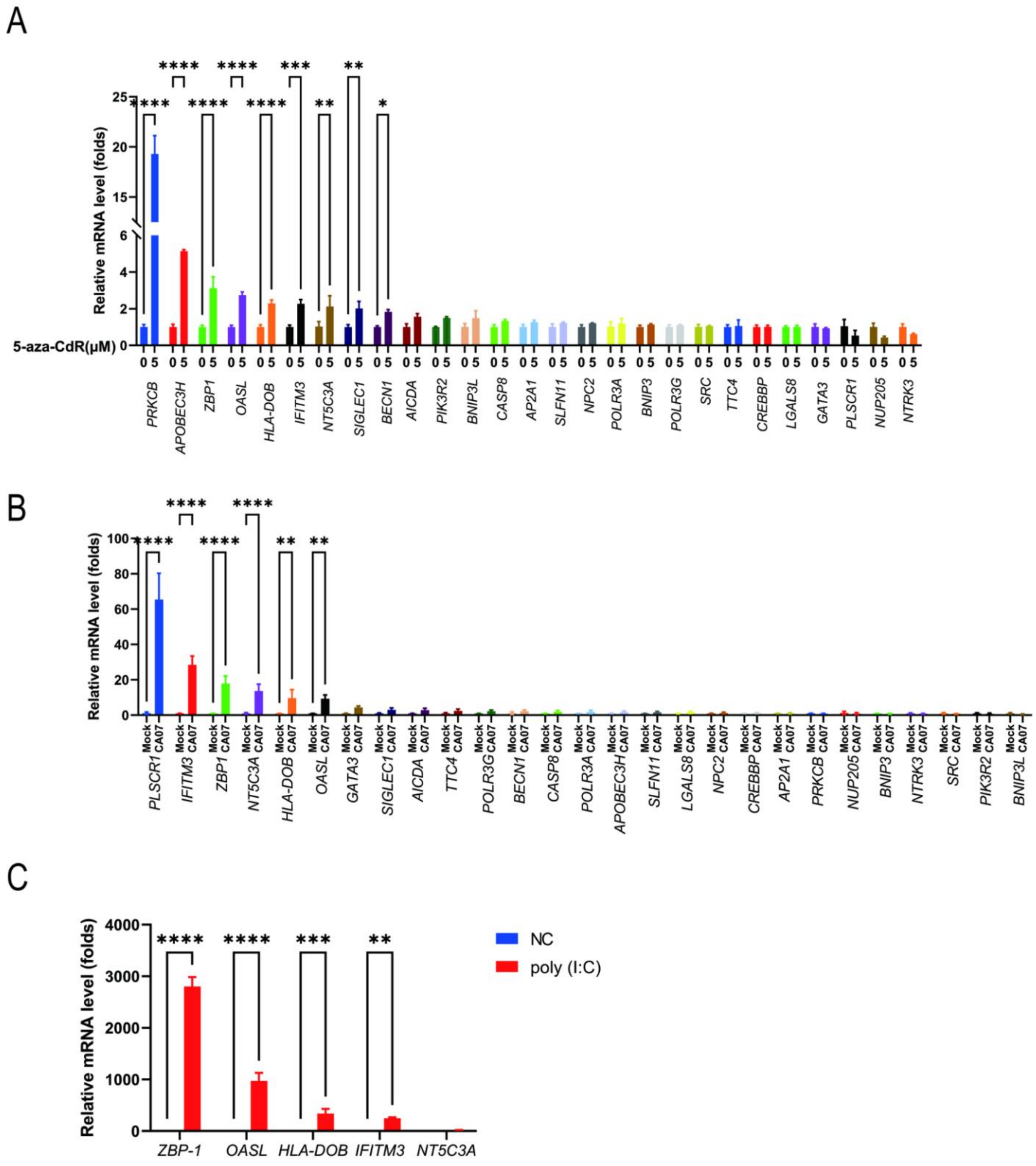

1.2 Supplementary Table

Supplementary Table 1. Sequences of primers used in the study.

Gene name	Forward	Reverse
DNMT1	CCAACAGAGGACAACAAGT	TGGTGGCTGAGTAGTAGAG
DNMT3A	GGACAAGAATGCCACCAA	TCCACCAAGACACAATGC
DNMT3B	AAGAGCAGCCTGGAAGAT	GTAAGACTGATAGCCATCGT
UHRF1	CATCATCAAGTGGCAGGA	CAGTCGTTCAGAGAATCATC
TET1	GCTCTGTTGCTACTGATATG	GATGGAGTGTGAAGAATGTG
TET2	GAACAGGATAGAACCAACCA	TCAGGACTCACACGACTAT
TET3	TGATCGTCATCCTCATCCT	GCAGCCGTTGAAGTACAT
β-actin	TGCGTGACATTAAGGAGAA	AAGGAAGGCTGGAAGAGT
IFN-α	CTTGATGCTCCTGGCACAGA	TCATGGAGGACAGGGATGGT
IFN-β	CATTACCTGAAGGCCAAGGA	CAATTGTCCAGTCCCAGAGG
RIG-I	GACCCTGGACCCTACCTACA	TCCATTGGGCCCTTGTTGTT
IRF3	CCTGCACATTTCCAACAGCC	AATCCATGCCCTCCACCAAG
IRF7	ATGGCCTTGGCTCCTGAGA	CGCTGCTGATCTCTCCAAGG
OASL	GTTCTCAGGAGCACCAGAGAGGT	GAAGACGAGAGCATCGGGGACT
SLFN11	CCTGGTTGTGGAACCATCTT	CTCTCCTTCTCTTGGTCTCTCT
APOBEC3H	AAGGCCCTCTTGTGTTACCAG	CACTGCGTTTCGTCCAGTC
BNIP3	CTGGACGGAGTAGCTCCAAG	CCGACTTGACCAATCCCATA

CASP8	AGAGTCTGTGCCCAAATCAAC	GCTGCTTCTCTCTTTGCTGAA
AP2A1	CTGCTTGGCATTGCTCCGGTCACAC A	TGGCCAGCTCCGAGTCTCCCATGA A
SIGLEC1	CCTCGGGGAGGAACATCCTT	AGGCGTACCCCATCCTTGA
NUP205	GAAACTTCTGGACATTGAAGGA	TGAGGATGGAACTAGGGGAAG
LGALS8	CCTTGCACTTTCCGGCAATC	GGGGGAGGTGTGAGCTACTA
PRKCB	CGTCCTCATTGTCCTCGTAA	TGTCTCATTCCACTCAGGGTT
BNIP3L	AGCAATGGCAATGATAATGG	CAACTTCTTCTTCTGACTGAG
BECN1	CCGTGGAATGGAATGAGAT	TGCCTCCTGTGTCTTCAA
PIK3R2	GGAGGAGGTGAACGAGAA	GGCGGTAGTGATTGATGAG
NT5C3A	CTCCAACAACATAGCATCC	ATTCCTCAAGGCACCATC
TTC4	CGTGAACTGGTGTGATGA	CATTCTGATTCCTCTCCTTCT
POLR3A	CCACCTCCAATAACACCTAT	GCACACTCTCCTTCATCTT
ZBP1	CCTCAGTTCAGCCAACAA	CAAGTCTCGGTTCACATCT
PLSCR1	GTATCCACCGACAGCATT	CCAGAGTTATGACTTCTTGAC
IFITM3	GGACAGGAAGATGGTTGG	TGACGATGAGCAGAATGG
NTRK3	GCCAACCAGACCATCAAT	CGAAGAGAACCACCAACA
POLR3G	AAGGCACACCACTCACTA	ATCATCGTCATCGTCATCAT
SRC	CAGGCTGAGGAGTGGTAT	GCTTGCGGATCTTGTAGT
GATA3	CACCACAACCACACTCTG	GCCTTCCTTCTTCATAGTCA

NPC2	GTCACCTTCACCAGCAAT	TCCACTCTTACAACCATCAG
HLA-DOB	GGCTCTGCTAGTGAATCTG	CTGTCTGCTCCTCTCCAA
AICDA	TGGACAGCCTCTTGATGA	GTCACGCCTCTTCACTAC
CREBBP	CAACCCCAAAAGAGCCAAACT	CCTCGTAGAAGCTCCGACAGT
Primers used for PCR prior to DNA methylation analysis		
Sequence NO.	Forward	Reverse
OASL.1	aagtagYtggggYtaYaggtggaYaYYa	tatcttaaatccatacctcttccctRaa
OASL.2	tgtggagtggggagttggataYatgagt	caacttccacattttaattttattttta
OASL.3	tgggYgagaYagtgagaYtYYatYtYaa	aaaaaaRcatRaaaacatRaaRccRRca
OASL.4	gYaggtgtYtgYtaaYaYYaaatgaaaYt	cctcccacaRtRctaRRattacaRcRtR
OASL.5	gaatYataataYYtgttaaaYYYaYtYt	acRRRttcaaRcRattctcctRcctca
OASL.6	acatgtctgtagtcccagctactaggga	agaacagactgggaattctagtctacct
OASL.7	aYtYtatgaatagtgagggtYaaagagg	RtcatcaRtttcRattctcaacttttaRaR
OASL.8	tttggggtYaaaYaYtatYYaaYttYagt	ctRRRcaRatatataRccaRRctcctac
Sequences of the siRNAs		
Gene name	sense（5'-3'）	antisense（5'-3'）
si-DNMT1	GCCGAAUACAUUCUGAUGGAUTT	AUCCAUCAGAAUGUAUUCGGCTT
si-OASL	GAAGACGAGAAUUUCAUGUTT	ACAUGAAAUUCUCGUCUUCTT
si-ctrl	UUC UCC GAA CGU GUC ACG UTT	ACG UGA CAC GUU CGG AGA ATT
